# Enhancement of Osseointegration via Endogenous Electric Field by Regulating the Charge Microenvironments around Implants

**DOI:** 10.1002/adhm.202403388

**Published:** 2025-01-05

**Authors:** Fangfang Xu, Guangbin Zhao, Yuxin Gong, Xiang Liang, Ming Yu, Hao Cui, Linyang Xie, Nan Zhu, Xuan Zhu, Xiaoxi Shao, Kun Qi, Bingheng Lu, Junbo Tu, Sijia Na

**Affiliations:** ^1^ Key Laboratory of Shaanxi Province for Craniofacial Precision Medicine Research College of Stomatology Xi’ an Jiaotong University Xi’ an 710004 China; ^2^ Clinical Research Center of Shaanxi Province for Dental and Maxillofacial Diseases College of Stomatology Xi'an Jiaotong University Xi’ an 710004 China; ^3^ Department of Oral and Maxillofacial Surgery College of Stomatology Xi'an Jiaotong University Xi'an 710004 China; ^4^ State Key Laboratory for Manufacturing System Engineering School of Mechanical Engineering Xi'an Jiaotong University Xi'an 710054 China; ^5^ State Key Laboratory of Oral & Maxillofacial Reconstruction and Regeneration National Clinical Research Center for Oral Diseases Shaanxi Clinical Research Center for Oral Diseases Department of Oral and Maxillofacial Surgery School of Stomatology The Fourth Military Medical University 145 West Changle Road Xi'an 710032 China; ^6^ Department of Orthodontics College of Stomatology Xi'an Jiaotong University Xi'an 710004 China

**Keywords:** charge microenvironment, endogenous electric field, osseointegration, stem cell differentiation

## Abstract

The regulation of the charged microenvironment around implants is an effective way to promote osseointegration. Although homeostasis of the charged microenvironment plays an integral role in tissues, current research is externally invasive and unsuitable for clinical applications. In this study, functional materials with different surface potential differences are prepared by changing the spatial layout of Ta and Ag on the surface of a Ti‐6Al‐4V alloy (TC4). This naturally formed an endogenous electric field (EEF) with a negatively charged cell membrane after in vivo implantation and promoted osseointegration at the interface between the bone and implant through the upregulation of Ca^2+^ concentration and activation of subsequent pathways. Interestingly, the promotion of stem cell differentiation, regulation of the direction of immune cell polarization, and antibacterial efficacy are determined by the free charge contained in the implant, rather than by the magnitude of the surface potential difference. This functional implant represents a unique strategy for regulating the charged microenvironment around the implant and enhancing osseointegration, thereby providing ideas and technical approaches for the clinical development of novel implant materials.

## Graphical abstract

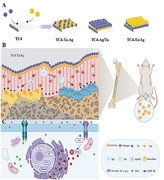

## 1 Introduction

With the aging of the modern population and frequent occurrence of traffic accidents, bone diseases, and injuries have significantly increased, leading to an urgent need to develop artificial implants with efficient bone integration capabilities to relieve pain and improve function in individuals with osteoarthritis and other damaged bone tissues.^[^ ^1^, ^2^ ^]^ Osseointegration is a complex healing process that occurs at the interface between the implant and bone tissue. Previous studies have attempted to change the mechanical and biochemical properties of implants. Titanium and its alloys are widely used as implant materials because of their excellent mechanical properties and biocompatibility. However, its biological inertness, stress shielding, and poor antibacterial performance limit its further development.^[^ ^3^, ^4^ ^]^ Therefore, the surface properties of titanium are crucial for implant success. Various materials such as organics, hydrogels, cytokines, and metal ions (Ag, Zn, Ta, Cu, and Mg) have been applied to modify the surface of titanium and its alloys through various strategies to enhance angiogenesis or osteogenesis and promote osseointegration.^[^ ^2^, ^5^, ^6^ ^]^ However, these strategies involve complex and laborious synthetic steps. They are poorly biocompatible, may cause unpredictable side effects, and have poor prospects for clinical applications. Moreover, an implant is a foreign body to the host tissue; therefore, success in vitro may not yield good results in vivo.^[^ ^7^ ^]^ The inconsistencies between the in vitro and in vivo experiments are mainly due to the insufficient consideration of the overall implanted microenvironment. Hence, designing functional implants based on an in‐depth understanding of osseointegration to regulate the microenvironment surrounding the implant is a more effective strategy.

Recent advances have focused on the regulation of the immune microenvironment, and this has improved the osseointegration performance to some extent.^[^ ^8^, ^9^, ^10^ ^]^ A better understanding of the microenvironment not only includes the immune microenvironment but also the bioelectrical microenvironment. Bioelectrical properties of living organisms are necessary for embryonic development and tissue repair.^[^ ^11^ ^]^ Bioelectrical signals play an important role in the communication between cells, tissues, and organs. Similar to piezoelectricity and electrostatics, it affects the arrangement and aggregation of macromolecules outside the cell and influences cell behavior in a biophysical way.^[^ ^12^ ^]^ The cell membrane consists of a phospholipid bilayer with each molecule having a hydrophilic head and hydrophobic tail. The head usually has a negative charge, and this electrical property of the cell membrane is important for the cell to maintain ion balance and transmembrane potential difference. When an implant is placed into the bone, its surface charge may differ from that of the surrounding cell membrane. This charge difference causes an EEF to form between the implant and the cell membrane. This electric field can guide ions such as Ca^2+^ from the surface of the cell to the surface of the implant and deposit them to form a mineralized layer similar to bone. EEF may promote bone formation by regulating the proliferation and differentiation of osteoblasts.^[^ ^13^, ^14^ ^]^ Bones exhibit unique electric cues derived from the piezoelectric collagen fibers of bones.^[^ ^15^ ^]^ Thus, regulating the bioelectric balance in the implant microenvironment may be an innovative strategy. Moreover, an exogenous electrical stimulation could interfere with the electric microenvironment of the cell, triggering a series of unique biological effects, like calcium fluctuation.^[^ ^2^, ^11^, ^16^, ^17^ ^]^ However, the development of biomaterials with an intrinsic in situ bone microenvironment remains challenging.

**Figure 1:**
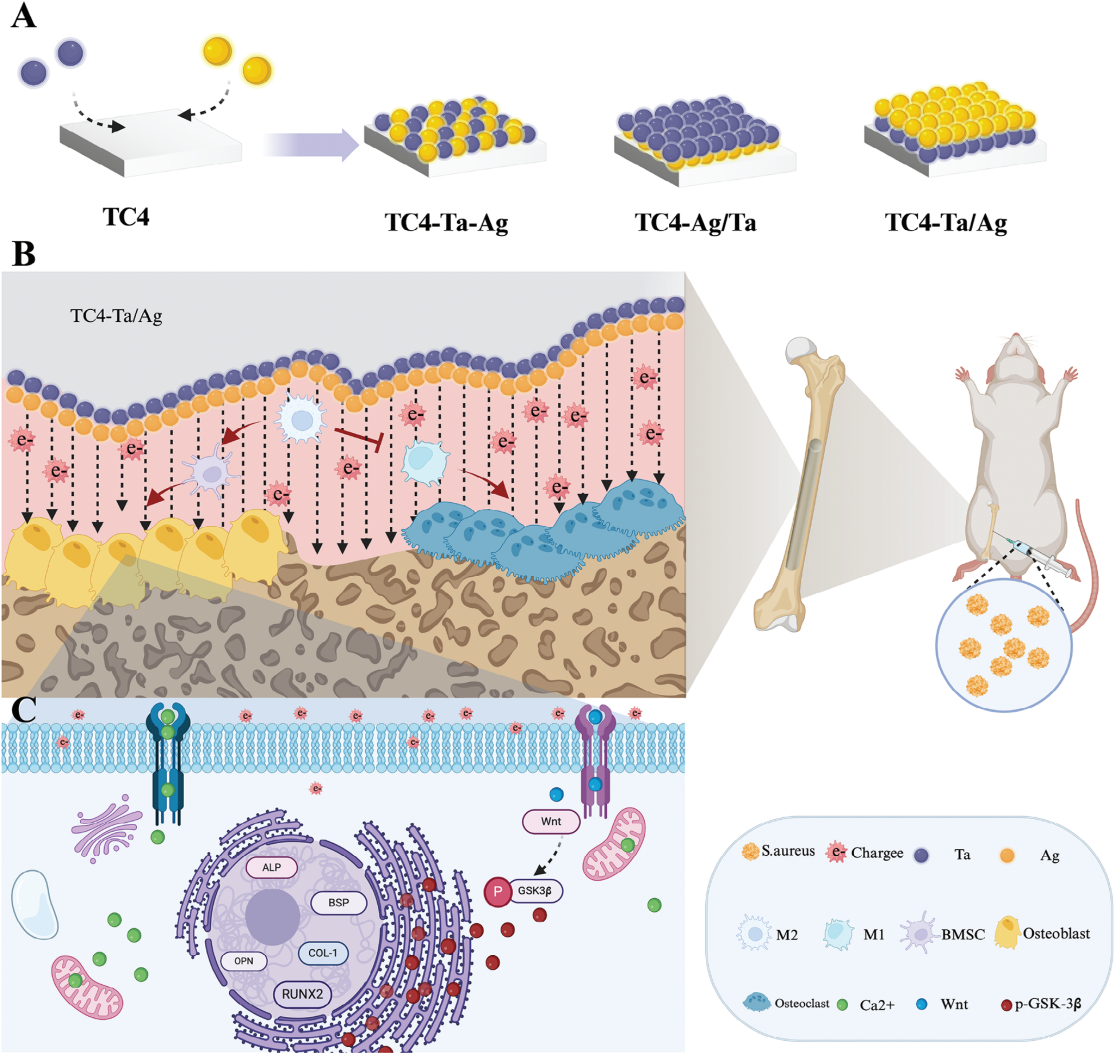
Schematic of EEF Regulation of Voltage‐Gated Channels to Promote Osseointegration. A),Functional materials with varying surface potential differences were fabricated by depositing Ta, Ag nanoparticles onto TC4 surfaces using magnetron sputtering, creating distinct spatial configurations. B), After in vivo implantation, an EEF is naturally formed which regulates macrophage polarization to the M2 phenotype and enhances antimicrobial efficacy. This EEF subsequently promotes the osteogenic differentiation of stem cells, thereby improving osseointegration capacity. C), By forming an EEF with negatively charged cell membranes, changes in the charge microenvironment led to alterations in the conformation of voltage‐gated ion channels. This, in turn, increased cellular Ca^2+^ uptake, activated the Wnt signaling pathway, and promoted the expression of osteogenesis‐related proteins.

To date, titanium‐based implants with long‐term electrically coupled signals have remained elusive.^[^ ^1^, ^18^ ^]^ In view of the EEF, we propose the regulation of the bioelectrical microenvironment in which the EEF, generated by manipulating the surface potential of the implant material, enhances osseointegration. To construct a functional implant with regulatable EEF‐mediated, Ta ions with excellent osteogenic properties and long‐term chemical stability and Ag ions with broad‐spectrum and efficient antimicrobial properties need to be incorporated. Therefore, we designed TC4 with Ta‐Ag, whose surface potential can be finely determined using different sputtering sequences. In vivo, an EEF was established between the positively charged implant and negatively charged cell membrane to guide directional osteogenic and antibacterial ions to promote osseointegration. Our findings (**Figure** **1**) highlight the potential for enhancing osseointegration by modulating the in vivo microenvironment, offering novel ideas and technical approaches for the clinical development of advanced implant materials.

## 2 Results

### 2.1 Morphological and Structure Characterization of TC4‐Samples

**Figure 2:**
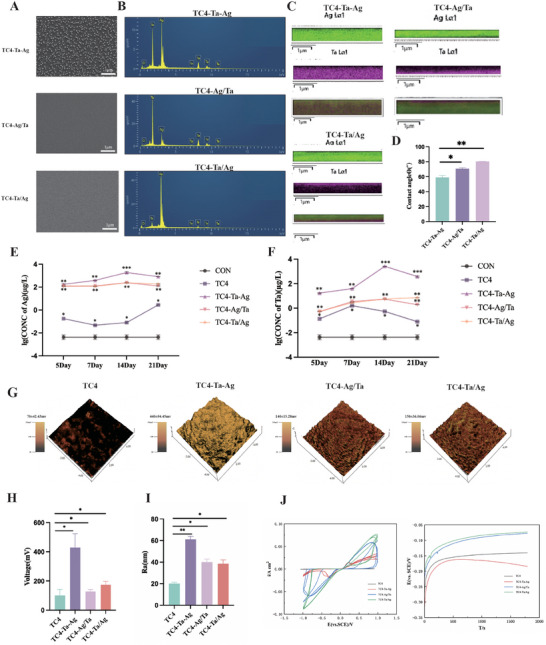
Sample fabrication and characterization A), SEM images of three groups. B), The Surface Element Distribution of TC4‐Ta‐Ag, TC4‐Ag/Ta and TC4‐Ta/Ag. C), Fault Energy‐dispersive X‐ray spectroscopy elemental map of the TC4‐Ta‐Ag, TC4‐Ag/Ta and TC4‐Ta/Ag, mappings of TA or Ag. D), Water contact angle of the groups evaluated. E, F), Ta and Ag ion release concentrations. G), SKPM characterization of the TC4‐groups. H, I), Roughness and Surface potential of TC4, TC4‐Ta‐Ag, TC4‐Ag/Ta and TC4‐Ta/Ag. J), CV and corrosion potential curve of TC4, TC4‐Ta‐Ag, TC4‐Ag/Ta and TC4‐Ta/Ag.(Green‐Ag, Purple‐Ta; ^***^ *P* < 0.001, ^**^ *P* < 0.01, ^*^ *P* < 0.05). These results confirm the successful incorporation of Ta and Ag ions to construct different surface electrostatic potential on TC4 implant.

To construct an electro‐microenvironment on the TC4 implant, Ta and Ag nanoparticles were deposited onto its surface by magnetron sputtering^[^ ^19^ ^]^ (Figure 1A). SEM images of the metal particles displayed a uniform microstructure (**Figure** **2**A). The elemental distribution was further verified by EDS mapping to confirm the successful deposition of Ta and Ag ions on TC4 (Figure 2B; Table S1, Supporting Information). A tomographic energy spectrum analysis revealed that materials were synthesized with different sputtering sequences, namely Ta and Ag co‐sputtering (TC4‐Ta‐Ag), Ag sputtering followed by Ta sputtering (TC4‐Ag/Ta), and subsequent Ag sputtering of Ta (TC4‐Ta/Ag) (Figure 2C; green: Ag and purple: Ta).

In addition to the surface elemental analysis, the surface wettability and roughness of the samples were further characterized by water contact angle measurements and AFM, respectively. The results of the experiments confirmed that the water contact angles of TC4‐Ta‐Ag, TC4‐Ag/Ta, and TC4‐Ta/Ag are ≈59°, 63°, and 80°, respectively, all of which are less than 90°.^[^ ^20^ ^]^ Thus, the high hydrophilicity of the samples can be attributed to their high hydrophilicity (Figure 2D). ICP was used to determine the ion release concentrations of Ta and Ag at different time points in each group^[^ ^21^ ^]^ (Figure 2E,F). The TC4 group showed no significant release of Ta or Ag ions. As shown in Figure 2E,F, the rapid release of Ta and Ag ions was detected in the TC4‐Ta‐Ag, TC4‐Ag/Ta, and TC4‐Ta/Ag groups over 14 days, after which the release rate decreased slowly. The TC4‐Ta‐Ag group was significantly different from the other groups (*p* < 0.01). The TC4‐Ag/Ta and TC4‐Ta/Ag groups did not differ significantly in terms of incubation time. Figure S1A (Supporting Information) showed that all the coatings sputtered on the TC4 surface did not show any fracture breakage and the coatings were intact in all the experimental groups. Through nanoindentation experiments, the hardness of the resultant surfaces TC4‐Ag/Ta is significantly higher than the other two groups. Young's modulus results showed no significant difference between the two groups of coatings TC4‐Ag/Ta and TC4‐Ta/Ag, but both were significantly higher than TC4‐Ta‐Ag (Figure S1B, Supporting Information). Compared to the TC4‐Ta‐Ag group, the TC4‐Ta/Ag group and the TC4‐Ag/Ta group had strong mechanical properties, which may help to maintain the distribution of the free charge of the EEFs and minimize the damage when subjected to bone extrusion during implantation into the bone.

SKPM characterization of the TC4‐samples showed different roughness and surface potentials (Figure 2G,I). The TC4 substrate showed a relatively smooth surface morphology with a roughness (*R*a) of 20.00 ± 1.41 nm. After the sputtering of Ta and Ag ions, small but dense particles were observed on the sample surface; furthermore, the *R*a values increased, with those of TC4‐Ta‐Ag, TC4‐Ag/Ta, and TC4‐Ta/Ag being 61.00 ± 2.83, 40.00 ± 2.83, and 53.53 ± 3.54 nm, respectively. The surface morphology and properties of the sputtered material changed, which likely led to a change in the potential difference at the surface of the material compared to TC4.

### 2.2 Electrochemical Properties of Materials

To investigate whether materials with different sputtering sequences exhibit different potential differences, we investigated the electrochemical properties of TC4, TC4‐Ta‐Ag, TC4‐Ag/Ta, and TC4‐Ta/Ag. The SKPM AFM mode was used to observe the potential differences on the surface of the material (Figure 2G,H). Potential differences below 500 mv favor the stimulation of cell proliferation and differentiation.^[^ ^22^ ^]^ The results indicate that the potential difference on the surface of the TC4‐Ta‐Ag group (440 ± 94.45 mv) was significantly higher than that on the surfaces of the other groups. This may be due to the higher surface roughness of the TC4‐Ta‐Ag group. The magnitude of the capacitance represents the ability to store free charge. A reduction in the impedance value is conducive to the response of the material to electrical signals and faster transmission of electrical signals to cells, thus affecting the physiological activity of the stem cells.^[^ ^23^ ^]^ CV and corrosion potential curves (Figure 2J) suggest that no obvious electrochemical double layer (EDL) was observed on TC4,^[^ ^24^ ^]^ but the curves of TC4‐Ta‐Ag, TC4‐Ag/Ta, and TC4‐Ta/Ag exhibited typical EDL capacitive behaviors. The results confirmed that TC4‐Ta/Ag exhibited the highest capacitance among all experimental groups. In contrast, TC4‐Ta‐Ag exhibited the lowest capacitance among the sputtering configurations.

### 2.3 In Vitro Cytocompatibility

**Figure 3:**
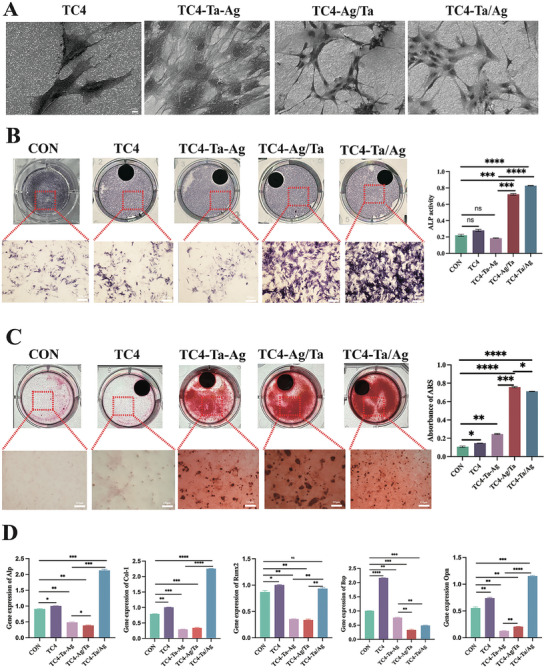
In vitro osteogenesis A), SEM images of BMSCs on different groups. B, C), ALP staining and ARS staining of BMSCs co‐cultured (after 7 days and 3 weeks) with different groups. D), Gene expressions of osteogenesis‐related proteins including COL‐1, ALP, Runx2, BSP, and OPN of BMSCs in different groups after 7 days of osteogenic induction (n = 3 per group; ^***^ *P* < 0.001, ^**^ *P* < 0.01,^*^ *P* < 0.05).

To verify the biocompatibility of the materials, BMSCs were inoculated on the surface of different materials. After five days of incubation, live‐dead cell staining was performed; no obvious dead cells were observed in any of the groups (Figure S2A, Supporting Information). Cytoskeleton staining and SEM were performed to observe the changes in cell morphology on the surface of the materials. The BMSCs tightly adhered to the surface of each group of materials (**Figure** **3**A; Figure S2B, Supporting Information). The cell morphology of the TC4‐Ta‐Ag group appeared crumpled, and the BMSCs in the TC4‐Ag/Ta and TC4‐Ta/Ag groups were tightly bound to each other and extended more than those cultured in these groups.

### 2.4 In Vitro Osteogenic Differentiation

An optimal bone implant material should possess osteoconductive and osteoinductive properties to promote bone integration.^[^ ^25^ ^]^ Therefore, we inoculated BMSCs onto the surfaces of different materials and performed ALP staining. As shown in Figure 3B, TC4‐Ag/Ta and TC4‐Ta/Ag exhibited better early osteogenic activity. After three weeks of co‐culture, the osteogenicities of the different groups were evaluated by ARS staining. The results showed that TC4‐Ag/Ta and TC4‐Ta/Ag significantly promoted osteoblast differentiation compared to the other groups (Figure 3C). To explore the osteogenic capacity of the BMSCs after co‐culturing with different materials, the expression of relevant osteogenic genes, including CoL‐1, ALP, Runx2, BSP, and OPN, was analyzed by qRT‐PCR. The COL‐1 and ALP gene expression results indicated that TC4‐Ta/Ag was significantly higher than that of the other groups (Figure 3D). However, the expression of BSP in the TC4‐Ta/Ag group was significantly lower than that in TC4 (*p* < 0.001), whereas the expression of OPN was upregulated, consistent with the trend of COL‐1 and ALP expression. This observation indicates that the upregulation of osteoblast‐related genes within TC4‐Ta/Ag persisted throughout the initial and terminal phases of osteogenesis.

**Figure 4:**
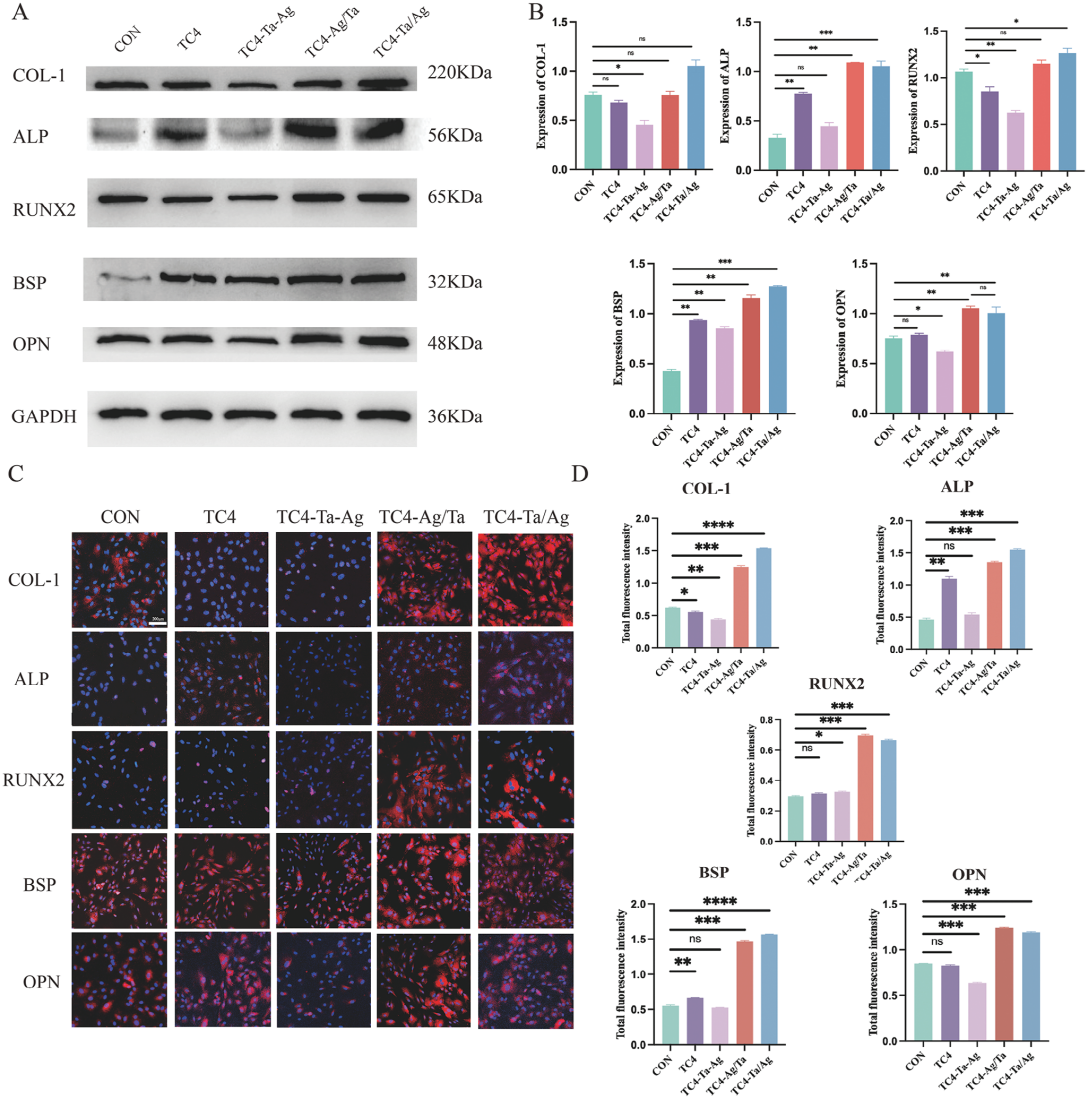
Expression of osteogenic related proteins A, B), Effect of inoculation on BMSCs protein expression (COL‐1, ALP, RUNX2, BSP, OPN) on the surface of different groups using WB after 14 days of osteogenic induction. C, D), Effect of inoculation on BMSCs protein expression on the surface of different groups using immunofluorescence. Mean values are shown and error bars represent ± s.d. (n=3 per group), as analyzed by a one‐way ANOVAs with post hoc Tukey Tests. (^***^ *P* < 0.001, ^**^ *P* < 0.01, ^*^ *P* < 0.05).

The differences in osteogenicity among the different groups were further verified by WB. As shown in **Figure** **4**A,B, the protein expression level of TC4‐Ta‐Ag was the lowest, and TC4‐Ta/Ag and TC4‐Ag/Ta were upregulated compared to the other groups, with the highest expression observed in TC4‐Ta/Ag, which proved to be the best at promoting osteogenicity. Immunofluorescence experiments showed that the expression of relevant osteogenic proteins, which was upregulated in TC4‐Ta/Ag and TC4‐Ag/Ta cells, was significantly higher than those in the other groups (Figure 4C,D). In summary, different sputtering sequences led to different potential differences on the material surface, which had different effects on the proliferation, differentiation, and osteogenic expression of BMSCs.

### 2.5 Whole‐Cell Patch‐Clamp Recording

**Figure 5:**
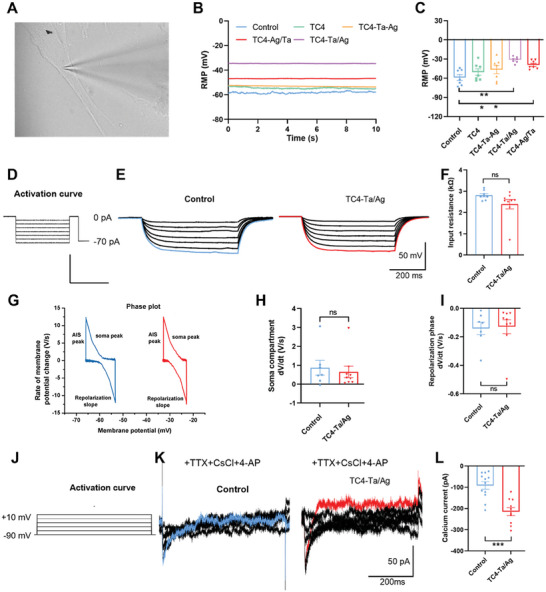
The characterization of electrical properties of TC4‐groups A), Images showing electrode tips clamped onto BMSCs, with tip resistances measured in the range of 3‐6 MΩ. B, C), The schematic and statistical graphs of RMP indicate that n exhibit significant differences compared to the control group (*P* < 0.01). D, E, F), The activation curve, schematic, and statistical graphs of input resistance indicate no significant difference between the control group and TC4‐Ta/Ag (*P*>0.05). G, H, I), Phase plot analysis showing no significant differences in the somatic and repolarization phases between the control group and TC4‐Ta/Ag group, indicating no changes in Na^+^ and K^+^ currents between the groups. J, K, L), Activation curves of calcium currents showing significant statistical differences in calcium currents between the control group and TC4‐Ta/Ag group after blocking Na^+^ and K^+^ currents.

The cell membrane acts as a barrier to the exchange of material and energy between the cell and the microenvironment of the organism via a process known as transmembrane transfer.^[^ ^26^ ^]^ Charge transfer is the primary pathway through which cells interact with the external environment. Generally, the movement of charge through the cell membrane creates an electrochemical gradient that affects the overall membrane potential.^[^ ^27^ ^]^ Therefore, a change in membrane potential indicates active or inactive charge movement. The whole‐cell membrane clamp technique was used to detect resting membrane potential (RMP) changes in different groups, as shown in **Figure** **5**A–C. The RMPs of the groups were −50.52 ± −8.02, −43 ± −14.09, −41.6 ± −18.43, −39.9 ± −7.02, and −30.8 ± −5.50 mv, respectively. The RMP in the TC4‐Ta/Ag group was significantly lower than that in the other groups (*P* < 0.01). However, the TC4‐Ta‐Ag group, which had a higher surface potential difference, showed no obvious change.

There was no statistical difference was observed between the input resistances of the control and TC4‐Ta/Ag groups (Figure 5D–F), suggesting that TC4‐Ta/Ag co‐culture with cells may enhance the excitation of cells by reducing the RMP, thus promoting their differentiation and maturation.^[^ ^28^, ^29^ ^]^ The changes in the RMP of cells are mainly related to the opening of Na^+^, K^+^, and Ca^2+^ ion channels in the cell membrane.^[^ ^30^, ^31^, ^32^ ^]^ To further explore the potential mechanism by which TC4‐Ta/Ag affects the RMP of the BMSCs, we first performed a phase‐plane analysis to detect the action potential (AP) waveform of the bone marrow stem cells. The analysis compared the relationship between the rate of voltage change and voltage during the AP. Experiments have shown that BMSCs are excitatory but do not release action potentials. Therefore, we recorded the subthreshold membrane potential of the BMSCs in the control and TC4‐Ta/Ag groups (Figure 5G). A statistical analysis showed that there was no significant difference in the rate of voltage change between the soma peak and repolarization slope of the somatic cells (Figure 5H,I), suggesting that the effect of TC4‐Ta/Ag on cell excitation may not have been caused by the changes in the Na^+^ and K^+^ channels.

Since the components associated with the axon initial stage (AIS) peak of the Ca^2+^ channels were not reflected in the subliminal membrane potential rise stage, Ca^2+^ channel changes were separately recorded after blocking the Na^+^ and K^+^ channels with TTX and CsCl/4‐AP, respectively. As the voltage gradient increased from −90 to + 10 mV, the calcium current amplitude increased gradually (Figure 5J,K). According to the statistical change of current at + 10 mV, the calcium current of TC4‐Ta/Ag co‐cultured cells (−215 pA) was significantly higher than that of the control group (−79 pA) (Figure 5L). These results suggest that TC4‐Ta/Ag may lead to the depolarization of BMSCs by affecting the opening of Ca^2+^ channels and increasing the intracellular Ca^2+^ concentration to promote the differentiation and maturation of cells.

### 2.6 Mechanisms of Osteogenesis In Vitro

**Figure 6:**
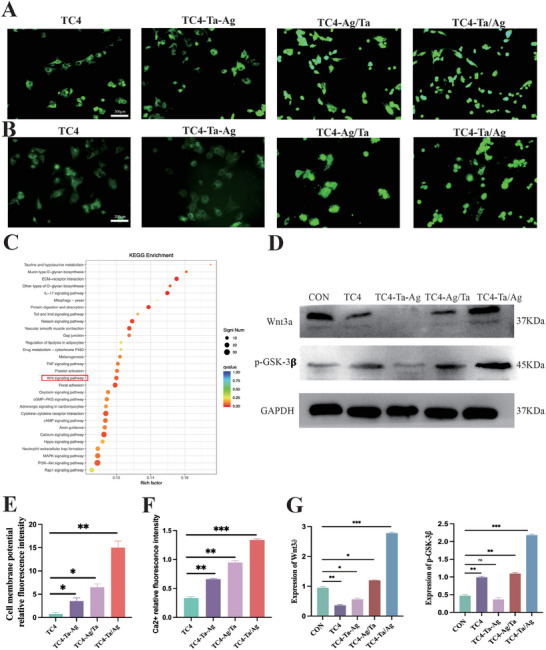
Osteogenic mechanism A, E), Changes in fluorescence intensity of membrane potential of cells inoculated on the surface of different groups. B, F), Changes in Ca2^+^ fluorescence intensity of cells inoculated on the surface of different groups. E: Representative top 20 upregulated pathways analyzed by the KEGG pathway method. C, D, G), Effect of inoculation on BMSCs protein expression (Wnt3a,p‐GSK‐3) on the surface of different groups usingWB. Mean values are shown and error bars represent ± s.d. (n=3 per group), as analyzed by a one‐way ANOVAs with post hoc Tukey Tests. (^***^ *P*<0.001,^**^ *P*<0.01,^*^ *P*<0.05).

To enhance the understanding of the molecular mechanism underlying charge‐induced osteogenesis, a membrane potential fluorescence probe was utilized. The probe indicated that TC4‐Ta/Ag had the most active membrane potential change. The fluorescence intensity of the TC4‐Ta‐Ag group was lower than that of the groups with different sputtering sequences, and TC4 was the weakest (**Figure** **6**A,E). The same change in calcium ion concentration was observed for the membrane potential (Figure 6B,F). Simultaneously, alterations in the calcium ion concentrations of TC4 and TC4‐Ta‐Ag were primarily observed in the cytoplasm (Figure 6B). However, the TC4‐Ag/Ta and TC4‐Ta/Ag groups exhibited changes not only in the cytoplasm but also in the nucleus (Figure 6B). Thus, TC4‐Ta‐Ag exhibited a smaller capacitance than the other two sputtering groups. This was due to the rapid release of metal ions into the liquid after entering the body fluid, which resulted in an inability to store sufficient free charges. Biologically, this was manifested by small changes in the membrane potential and calcium ions.

We sequenced the gene expression levels in BMSCs cultured from various samples. We then used a KEGG pathway analysis to identify the upregulated pathways. Figure 6C shows 20 representatives of upregulated signaling pathways. Notably, the Wnt signaling pathway, which regulates osteogenesis, was significantly upregulated, suggesting its involvement in the BMSC differentiation. A western blot analysis was performed to evaluate the proteins related to the integrin Wnt signaling pathway based on the KEGG pathway analysis results. The western blot results further revealed that the TC4‐Ag/Ta and TC4‐Ta/Ag groups exhibited increased relative expression of wnt3a and p‐GSK‐3𝛃 compared with the other group. However, the relative expression in the TC4‐Ta/Ag group increased more significantly (Figure 6D,G), indicating that the Wnt signaling pathway was activated.

### 2.7 In Vitro Immunomodulation

**Figure 7:**
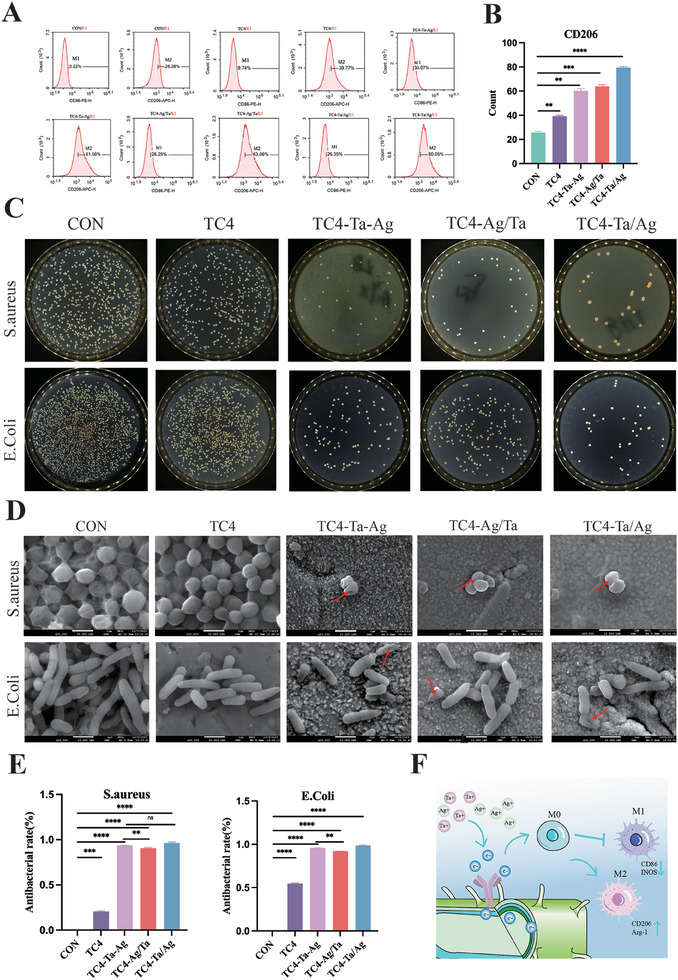
In vitro immune regulation and antibacterial A, B), Effect of inoculation on RAW264.7 polarization on the surface of different groups using Flow cytometry. C), E. coli and S. aureus (10^7^ mL^−1^) were incubated on different groups for 24h, resulting in solid cultures. D), FE‐SEM images of S. aureus and E. coli cultured on different surfaces are shown. The arrows indicate bacteria with damaged membranes. (scale bar = 5µm and 1µm, respectively). E), Antibacterial activity of different groups was determined by counting bacterial colonies. F), Schematic illustration of the mechanism on immunomodulation by the biocharge in a physiological environment. (Mean values are shown and error bars represent ± s.d. n=3 per group, as analyzed by a one‐way ANOVAs with post hoc Tukey Tests.^***^ *P*<0.001,^**^ *P*<0.01,^*^ *P*<0.05).xg500).

Next, we investigated whether materials with different surface potentials affect the polarization of macrophanes.^[^ ^17^ ^]^ Hence, we co‐cultured RAW264.7 with the material to see if cell polarization was modulated by materials of different surface potential groups. The RAW264.7 cells used were identified. An apoptosis assay demonstrated that macrophages exhibited excellent biosecurity (Figure S3A,B, Supporting Information). When LPS is added to the culture medium of RAW264.7, it stimulates various immune responses and inflammatory pathways within the cells, mimicking the response that occurs when the body encounters a bacterial infection or inflammation in vivo.^[^ ^33^ ^]^ Then, RAW264. 7 were co‐cultured with different materials for seven days. Experiments (Figure S3C,D, Supporting Information; **Figure** **7**A, B) showed that TC4‐Ta/Ag promoted the strongest macrophage polarization toward M2 type, and the expression of its M2 surface markers (CD206 and Arg‐1) was significantly up‐regulated, while the expression of its M1 surface markers (CD86 and INOS) was significantly lower in the other groups. This indicates that TC4‐Ta/Ag had the strongest anti‐inflammatory effect, while the expression of M2 surface markers by the TC4‐Ta‐Ag group was lower than the TC4 group, probably due to surface charge overload. Flow cytometry and immunofluorescence confirmed these results (Figure S3E,F, Supporting Information). TC4‐Ta/Ag, which enables more active changes in cell membrane potential, promotes polarization of RAW264.7 toward M2 and has excellent immune regulation ability.

### 2.8 In Vitro Antibacterial Activity

Infection remains one of the principal factors contributing to the inadequate osseointegration of bone implants.^[^ ^34^, ^35^, ^36^ ^]^ The antimicrobial properties of the materials were evaluated by co‐culturing them with S. aureus or E. coli and counting the number of colonies. As shown in Figure 7C, the TC4‐Ag /Ta, TC4‐Ta/Ag, and TC4‐Ta‐Ag groups inhibited bacterial growth better than the other groups. As shown in the SEM images, the bacteria in the CON and TC4 groups appeared smooth with clear edges. For the bacteria on TC4‐Ag /Ta, TC4‐Ta/Ag, and TC4‐Ta‐Ag, some membranes were damaged, exhibiting crumpling and deformation (Figure 7D). Bacterial live‐dead staining showed that the TC4‐Ag /Ta, TC4‐Ta/Ag, and TC4‐Ta‐Ag groups can significantly inhibit the proliferation of S. aureus and E. coli, as well as promote bacterial death, and the antimicrobial effect is significant (Figure S4, Supporting Information).

These results indicate that the functional implants designed by us effectively promote the antibacterial effect under the action of EEF, rather than just the release of antibacterial ions (Figure 7F).

### 2.9 In Vivo Osteointegration

**Figure 8:**
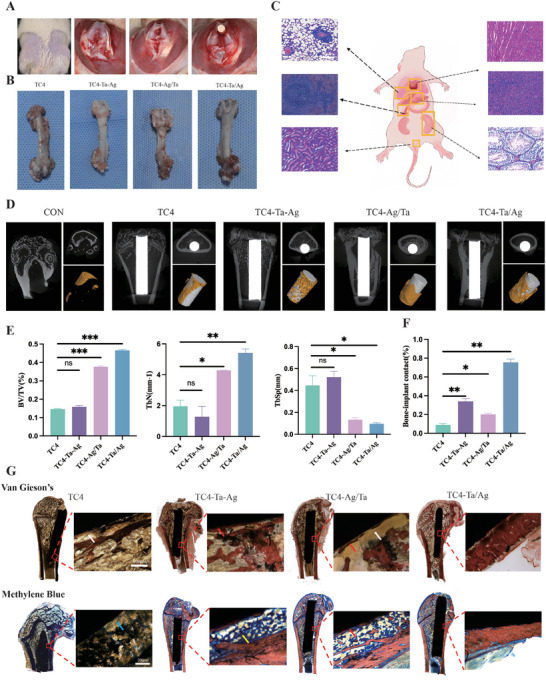
In vivo experiment to evaluate the antibacterial and osteogenesis properties. A), Establishment of implant‐related infection model. B), Gross view of the isolated femur after 8 weeks. C), Effects of bone implants on Heart, Liver, Spleen, Lungs, Kidneys and Testicles using H&E staining. D), Micro‐CT scanning of the femurs containing different implants and the reconstructed 3D images. E), Quantitative analysis of BV/TV, Tb⋅N and Tb. Sp. F,G), Effects of bone implants on BIC using VG staining and methylene blue staining. (n = 3 per group; ^***^ *P*<0.001,^**^ *P*<0.01,^*^ *P*<0.05).

Inconsistent in vitro and in vivo validation results may result from inadequate consideration of the overall implantation microenvironment.^[^ ^37^, ^38^ ^]^ To investigate the effect of the modified charged microenvironment on osseointegration, we conducted additional in vivo experiments (**Figure** **8**A,B). To observe whether the material was biologically safe in vivo, we isolated the heart, liver, spleen, lungs, kidneys, and testes and performed H&E staining. The results showed no obvious inflammatory cells or lesions under a microscope, suggesting that our material was not toxic to systemic organs (Figure 8C; Figure S5, Supporting Information). The femur with the implant was scanned by micro‐CT to assess the osseointegration ability of the material. Figure 8D shows cross‐sections in the longitudinal and transverse planes of femurs implanted with different bone implants and micro‐CT images after 3D reconstruction. The newly formed bone around the TC4‐Ta/Ag implants was denser than that observed in the other groups. With a bone volume/total volume of 45%, trabecular number exceeding 5.5 mm^−1^, and trabecular separation of 100 mm (Figure 8E), the TC4‐Ta/Ag group exhibited the best osseointegration performance. VG and methylene blue staining were performed to assess the inflammatory cells and bacterial infiltration in peri‐implant tissues (Figure 8G). After the implantation of TC4, the presence of fibers between the new bone and the implant prevented the new bone from coming into direct contact with the implant, resulting in fibrous wrapping. In the other three groups, trabeculae formed on the surface of the implants, and there was no fiber intervention between them. The largest bone‐to‐implant contact ratio was observed in the TC4‐Ta/Ag group (≈78%; Figure 8F).

The osseointegration of TC4‐Ta/Ag was superior because it allowed direct contact between the new bone and implant. However, TC4‐Ta‐Ag exhibited an even better osseointegration ability than TC4‐Ag/Ta owing to the rapid release of ions into the body, which had an anti‐inflammatory effect on S. aureus or E. coli, as shown in antibacterial results in vitro. Consequently, a small amount of new bone came into direct contact with the implant. TC4‐Ta‐Ag and TC4 bone implants formed a distinct fibrous envelope between the implant and the new bone. In summary, our strategy provides good biosafety and adequate in vivo antibacterial efficacy when Ta sputtering is followed by Ag sputtering on the TC4 surface. The large capacity of TC4‐Ta/Ag resulted in a high charge storage capacity, which generated an active action potential on the cell membrane, ultimately manifesting itself in the ability to improve osteointegration in the inflammatory environment in vivo.

## 3 Discussion

Osseointegration is defined as “the organized, direct connection between the living bone and surface of an implant.^[^ ^39^ ^]^” As foreign bodies, implants inevitably cause changes in the surrounding microenvironment. This paper describes biological implants with surface electrostatic potentials and highlights the advantages of such implants in biomedical applications. Specifically, by altering the spatial arrangement of the functional materials on the substrate surface, various electrostatic potentials can be generated, potentially facilitating the treatment of diverse diseases.

Do different spatial arrangements cause variations in the surface potential of TC4 implants? As illustrated in Figure 2G,H, different sputtering sequences of Ta and Ag on the TC4 surface resulted in varying surface potentials. Specifically, the TC4‐Ta‐Ag group exhibited a significantly higher surface potential difference compared to the other groups, with statistically significant differences observed. The cell membrane potential, generated by differences in the ion concentrations inside and outside the cell, plays a crucial role in cell signaling, metabolism, and the regulation of gene and protein expressions.^[^ ^40^, ^41^, ^42^ ^]^ Conducting materials with different potential differences were implanted via charge exchange with the cell. These inevitably caused a change in the membrane potential and thus regulated the biological behavior of the cell. In an in vitro membrane clamp recording assay, two questions were answered: 1) What are the characteristics of the changes in cell membrane potential induced by TC4‐Ta‐Ag, TC4‐Ag/Ta, and TC4‐Ta/Ag? 2) What is the potential mechanism for the changes in the implanted microenvironment? We found that both TC4‐Ag/Ta and TC4‐Ta/Ag significantly reduced the RMP of BMSCs without affecting the input resistance (Figure 5A–F), but the RMP of TC4‐Ta‐Ag was not statistically different from that of the control group. This finding contradicts the fact that TC4‐Ta‐Ag has a high surface potential difference. Because cell membrane potentials are generated by differences in ion concentrations inside and outside the cell, we conjectured that the higher surface potential difference of TC4‐Ta‐Ag might contain less free charge, leading to its inability to continuously keep the cell in a more excitable state; TC4‐Ta/Ag instead had more free charges, as demonstrated in Figure 2J. Do different spatial arrangements cause variations in the mechanical properties of implants? When a coated material is subjected to mechanical compression by the bone tissue after implantation, the compressive resistance of the coating determines whether it can maintain its electrical properties without structural changes. If the coating is strong enough to withstand the pressure exerted by the bone tissue, the surface charge distribution of the coating may not be significantly affected. However, if the resistance of the coating to compression is weak, mechanical stress may lead to deformation of the microstructure of the material, which in turn may alter its surface potential. As illustrated in Figure S1B (Supporting Information), the different sputtering sequences of Ta and Ag on the TC4 surface resulted in varying mechanical properties. The TC4‐Ta/Ag and TC4‐Ag/Ta groups had strong mechanical properties, which helped maintain the distribution of the free charge of the EEFs with minimal disruption when subjected to bone extrusion during implantation into the bone. In contrast, the TC4‐Ta‐Ag group was less influenced because of its lower mechanical properties compared to the other two groups. It is possible that after implantation, the distribution of free charges may be affected by each other. The next step of this study will be devoted to studying the relationship between the distribution of free charges of the implanted material coating and mechanical stress.

The opening of Na^+^, K^+^, and Ca^2+^ channels in the cell membrane is the most likely cause of the changes in the membrane potential.^[^ ^30^, ^31^, ^32^ ^]^ When trying to find exactly which ion channel caused the change in membrane potential, we found that TC4‐Ta/Ag did not trigger the AP, and there was no statistically significant difference in the rate of voltage change of the soma peak and repolarization slope compared with the control group. These results suggest that the excitatory effect of TC4‐Ta/Ag on cells may not have been caused by the changes in the Na and K^+^ channels. After the blocking of the Na^+^ and K^+^ channels, the intracellular calcium currents in the TC4‐Ta/Ag group were significantly higher than those in the control group, suggesting that osseointegration facilitated by implants with different spatial layouts may increase the intracellular Ca^2+^ concentration by the opening of Ca^2+^ channels and thus depolarizing the BMSCs.

Changes in the calcium ion levels play an important role in the initiation of MSC differentiation. The phospholipid bilayer of the cell membrane is a natural capacitor; when the difference between the inside and outside of the membrane reaches a certain domain value, the phospholipid bilayer becomes a continuous channel for the exchange of ions inside and outside the membrane, which affects the corresponding biological behavior of the cell.^[^ ^43^ ^]^ Changes in the charged microenvironment (Figure 6A–D) led to changes in the conformation of the voltage‐gated ion channels, which in turn increased the cellular uptake of Ca^2+^, activated the Wnt signaling pathway, and promoted the differentiation of stem cells.

An implant is a foreign body, and the cells recruited from the implant are in a microenvironment characterized by ischemia, hypoxia, and inflammatory stress, which can lead to the formation of fibrotic tissue. If macrophages are in the M1 phenotype for a longer period, they fail to polarize to the M2 phenotype in a timely manner. Thus, the balanced polarization of M1/M2 type macrophages plays an important role in this process.^[^ ^37^, ^38^, ^44^ ^]^ Altering the charged microenvironments around implants causes corresponding changes in the macrophages, yet there are few studies regarding this. In this experiment, we found that the TC4‐Ag/Ta and TC4‐Ta/Ag groups with higher surface potential differences downregulated the expression of CD86 and INOS and upregulated the expression of CD206 and Arg‐1, which promoted macrophage polarization toward the M2 type. This finding confirms the involvement of the charged microenvironment in regulating macrophage polarization and suggests that implants with higher potential differences have a greater effect on polarization. However, TC4‐Ta‐Ag, which possesses a higher surface potential difference, can promote macrophage polarization toward the M2 type. In contrast, TC4‐Ta/Ag with a relatively lower potential difference, performs more moderately. Hence, we hypothesized that it is related to the lower amount of free charge in TC4‐Ta‐Ag, despite its higher ion release, which may have influenced cell polarization. However, relative to that induced by the change in charge, the effect is relatively weak, and the specific related mechanism remains to be further investigated.^[^ ^8^, ^45^, ^46^ ^]^

Once the implant is infected, the layer of biofilm that forms between the surface of the material and tissue not only provides a platform for the proliferation of bacteria but also prevents the penetration of antibacterial drugs, thus causing loosening or even falling off of the implant, which is one of the most serious complications after surgical implantation.^[^ ^47^ ^]^ Cell membranes are negatively charged, and once they encounter a positively charged implant surface, an EEF naturally forms between them.^[^ ^13^, ^48^ ^]^ This study demonstrates the role and potential mechanisms of EEF in osteogenesis and immune regulation. Can the EEF generated by our implants disrupt biofilms, thereby acting as an antimicrobial agent? In this study, TC4‐Ta‐Ag, TC4‐Ag/Ta, and TC4‐Ta/Ag were co‐cultured with bacteria, and we found that implants with different surface potentials could significantly inhibit bacterial proliferation (Figure 7C–E). Notably, the antibacterial effect of the TC4‐Ta‐Ag group with a higher surface potential difference was not better than that of the TC4‐Ta/Ag group in vitro or in vivo (Figures 2E,F and 8D–G). This was probably because TC4‐Ta‐Ag actively releases antibacterial ions at a faster rate than TC4‐Ta/Ag within a short period of time, but with the release of ions, EEF weakens, resulting in a weakened antibacterial effect.^[^ ^49^ ^]^ Therefore, we can infer that the antibacterial effect of the implants was improved by EEF (Figure 7F).

Our research highlights the potential of charged microenvironments in biological organisms. By strategically altering the spatial layout of Ta and Ag on the surface of the TC4 cells, we formed an EEF that interacted with negatively charged cell membranes. EEFs naturally regulate macrophage polarization toward the M2 phenotype, thereby enhancing antimicrobial efficacy. From a mechanistic perspective, EEF changes the conformation of voltage‐gated ion channels, which in turn increases the cellular uptake of Ca^2+^, activates the Wnt signaling pathway, and upregulates osteogenesis‐related genes and proteins, including COL‐1, ALP, RUNX2, BSP, and OPN, thereby promoting stem cell differentiation. Consequently, the EEF promoted the osteogenic differentiation of stem cells, improving the overall osseointegration capacity of the implants.

Ultimately, our findings open new avenues for the development of advanced implant materials with intrinsic bioelectrical properties, aimed at improving the clinical outcomes of patients with osteoarthritis and other bone‐related injuries. This innovative approach not only offers a promising strategy for better osseointegration, but also paves the way for the next generation of functional biomaterials that can significantly interact with their biological environment.

## 4 Conclusion

In summary, this study provides a Ta‐ and Ag‐ion implant for constructing an EEF with a negatively charged cell membrane to study the effect of the EEF on osseointegration. This study demonstrates that the EEF regulates bone integration by regulating the opening and closing of Ca^2+^ ion channels, controlling the flow of calcium ions, and promoting bone integration at the bone‐implant interface. Therefore, regulating bioelectric balance in the implant microenvironment may be a useful strategy. These findings highlight the potential for enhancing osseointegration by modulating the in vivo microenvironment, offering novel ideas and technical approaches for the clinical development of advanced implant materials. Since the role of EEF was discussed in this study on a two‐dimensional plane and the implantation site is a complex multidimensional structure, subsequent studies will focus on how the temporal and spatial changes in micro‐EEFs formed by the multidimensional spatial layout of metal ions regulate the voltage‐gated calcium ion channels and their effects on osteogeny.

## 5 Experimental Section

### Preparation of Specimens

The medical‐grade Ti‐6Al‐4V alloy (TC4, Beijing, China) was cut into disk‐shaped specimens (diameter = 10 mm, thickness = 1.5 mm) for in vitro experiments and cylindrical specimens (diameter = 2 mm, length = 10 mm) for in vivo applications. Prior to surface treatment, all TC4 substrates were ultrasonically cleaned in acetone, ethanol, and deionized water for 10 min each, then air‐dried and mounted on the substrate holder of a Discovery635 magnetron sputtering system (China). High‐purity tantalum (Ta) and silver (Ag) targets (99.99% pure, diameter = 76.2 mm) were used for sputtering, and the substrates were maintained at room temperature. The target current was set at 0.3 A with a constant argon (Ar) flow rate of 20 sccm. Ag was sputtered first for 10 min, resulting in a 0.25 mm thick film, followed by Ta sputtering for 18 min to form a 0.25 mm thick Ta layer. Finally, a Ta‐Ag co‐sputtering process was carried out for 12 min, yielding a 0.5 mm thick combined film. All deposition steps were carefully monitored to ensure uniform film thickness and surface coverage.

### Characterization of Specimens

The surface morphology of the sample was characterized by field‐emission scanning electron microscopy (FE‐SEM, JSM‐7900F, JEOL, Japan). Energy dispersive X‐ray spectrometry (K‐Alpha, Thermo Electronics, USA) was used to determine the elemental composition and distribution of the samples. The roughness of the samples was conducted by an atomic force microscope(SHIMADZU, JAPAN). Water and diiodomethane contact angles of samples were determined by contact angle measurement (KRUSS, DSA100, Germany). Ta and Ag ions were measured using an inductively coupled plasma mass spectrometer (NexION 350D ICP‐MS, PerkinElmer, United States).

The electrochemical properties of the samples were characterized using a three‐electrode system on an electrochemical workstation (Corrtest, Wuhan, China). Measure the open‐circuit potential for 30 min first and CV was carried out from −1 to 1 V at a scanning rate of 100 mV s^−1^ with a sweep of 3 revolutions. The surface potentials of the samples were characterized by scanning Kelvin probe force microscopy (SKPM, SHIMADZU, JAPAN).

### Cell Culture

Bone marrow MSCs were isolated from the bone marrow fluid of SD male rats. Cells were cultured in α‐MEM medium with 10% fetal bovine serum (FBS, Gibco). Subsequent experiments were performed analogously with P3 generation cells on the 48‐well plate. BMSCs co‐cultured with TC4, Ta‐Ag, Ag/Ta, and Ta/Ag, at a density of 1  ×  10^4^ cells per well. The samples were sterilized under ultraviolet light for 2 h before cell seeding. The cells were cultured with the medium refreshed every two days and observed by a Zeiss light microscope to ensure the attachment of cells to the scaffold.

### Cytocompatibility

After culturing BMSCs on different samples for 7 days, a calcein‐AM/PI Double Stain Kit (Solarbio Science & Technology Co, Beijing, China) was applied to stain the dead and live cells. Cells in red, LDH, and CCK‐8 kits (Boster, China) were also used for the determination of proliferation and cytotoxicity of BMSCs cultured on different samples. Briefly, the absorbance at 450 nm was measured on a full‐wavelength plate reader (Synergy H1, USA) after 200 µL of 10% CCK‐8 solution was added to each sample (Bosterbio, China) after 1.5 h of incubation.

### Cell Adhesion and Morphology

The BMSCs cultured on the different samples were also detected by fluorescence staining. In brief, after 72 h of cell culture, the cells were fixed on different fixed with 4% paraformaldehyde for 30 min at 4 °C, and 0.1% Triton X‐100 (Solarbio Science & Technology Co, Beijing, China) was used to permeabilize the cells for 10 min. Then Alexa FluorTM 488 phalloidin (CA1620, Solarbio, Wuhan, China) was added and incubated for another 1 h in the dark. The cells were then counterstained with DAPI (Solarbio, China), followed by PBS, and observed under a laser confocal microscope (Olympus, JAPAN). SEM was also performed to examine cell adhesion and morphology. After 5 days in co‐culture, the cells were fixed with glutaraldehyde (2.5% v/v) for 2 h and dehydrated in ethanol gradients from 20%(V/V) to 100%(V/V). The samples were immersed in isoamyl acetate and critical‐point dried. The prepared samples were imaged using SEM (Flexsem1000, S‐4700, HITACHI, Japan).

### Intracellular Calcium Levels

BMSCs seeded on the 48‐well plate with different samples for 48 h were used to measure intracellular calcium. Intracellular calcium levels in cells were measured with Fluo‐3 calcium assay kits (Solarbio, China) according to the manufacturer's instructions. Briefly, after removing the medium, 25 µL Fluo‐3 was added to each sample and incubated at 37  °C for 60 min. After washing with PBS, the cells were observed by a fluorescence microscope.

### Matrix Mineralization

After 7 days of osteogenic induction, ALP staining, and Alizarin Red Staining were performed. Cells were washed three times in PBS and then fixed in 4% paraformaldehyde (26 °C, 10 min). For ALP staining, the cells were incubated in a BCIP/NBT working solution (Beyotime, China) for 20 min in the dark. For Alizarin Red Staining, the cells were fixed with 4% w w^−1^ paraformaldehyde for 15 min, and 40 mM Alizarin red S (ARS, Solarbio, China) was added for staining (26 °C, 30 min). Subsequently, the cells were washed 3 times with deionized water and observed by optical microscopy (TMS, Nicon, Germany).

### qRT‐PCR

After co‐culturing with different samples for 7 days, the cell media was removed before the addition of the Trizol reagent. After extraction, RNA samples were treated with RNase‐free DNase I (AG, China). They were then reverse transcribed into cDNA using a synthesis kit (AG, China). The gene levels of ALP, Runx2, COL1A1, BSP, OPN, and OCN were analyzed by qRT‐PCR (AG, China) using a mixture of dNTP reagent (AG, China) and RNase‐free H2O (AG, China), as well as the forward and reverse primers listed below. The 2ΔΔCT method was used to analyze gene expression data. The primer sequences are listed in Table S2 (Supporting Information).

### Western Blots

RAW264.7 and BMSCs co‐cultured with different samples for 14 days at a density of 4 × 10^5^ cells wel^−1^ in the 6‐well plates. The proteins were further quantified using the BCA kit. The samples with equal amounts of proteins (20 µg) were then separated by SDS‐PAGE gel. Subsequently, 5% skimmed milk was used to block the PVDF membranes with transferred proteins. After incubation with primary antibodies, including COLIA1 (1:1000), ALP (1:1000), RUNX2 (1:1000), BSP (1:1000), OPN (1:1000), CD86 (1: 10000), CD206(1:10000), INOS (1:10000), Arg‐1(1:10000), Wnt3a(1:10000), p‐GSK‐3𝝱(1:10000) and GAPDH(1: 1000) overnight at 4 °C, the PVDF films were incubated with anti‐rabbit IgG, HRP‐conjugated antibody (1:2000) for a further 1.5 h. Chemiluminescence detection was performed to visualize protein bands using ECL Substrate. The source data file contains untrimmed and unprocessed full‐scan images of all Western blots.

### Cell Membrane Potential Detection

Changes in the cell membrane potential can be reflected by changes in fluorescence intensity. Briefly, co‐culture on the 48‐well plate for 24 h, digest the cells from the surface of the material to obtain a cellular precipitate. Add DiBAC4 (3)(Solaibio, China)and control concentration range (1–5µmol L^−1^). The change in fluorescence intensity after stimulation was observed using a fluorescence microscope (PE‐300, Germany).

### Whole‐Cell Patch Clamp

The BMSCs (3 × 10^3^ cells cm^−^^2^, Neurobasal medium) were co‐cultured with each group for 3 days before being used for electrophysiological experiments. Data were collected using an Axon 700 B amplifier (Axon Instruments, CA).

RMP: cells were voltage‐clamped at −70 mV. A step command from −70 to 0 pA with a duration of 500 ms was applied, allowing the recording of voltage changes in response to the current. Input resistance was calculated using the formula:

(1) $$\mathrm{Resistance}=\frac{\mathrm{Voltage}}{\mathrm{Input}\;\mathrm{current}}$$

Phase Plane Analysis: An inward current pulse with an amplitude of 5 pA was applied, with a step command ranging from 0 to 200 pA and a duration of 15 ms. The subthreshold membrane potentials of the cells in the control group and the TC4‐Ta/Ag group were recorded at 200 pA. Phase plane plots were generated, and the rate of voltage change during the somatic and repolarization phases was statistically analyzed.

To detect inward calcium currents, CsCl was added to the internal electrode solution. TTX (1 µM) and 4‐AP (1 mM) were sequentially added to the perfusion solution to block Na^+^ and K^+^ currents. Cells were voltage‐clamped at −70 mV, and a step command ranging from ‐90 to +10 mV with a duration of 500 ms was applied. After stabilizing the whole‐cell configuration for 5 min, ion channel currents at different voltage conditions were recorded (Pclamp11.02 software).

### Immunofluorescent Staining

The BMSCs co‐cultured with different groups After 14 days and RAW264.7 co‐cultured with different samples for 5 days were fixed with paraformaldehyde (4% w w^−1^) at 4 °C for 30 min. Then, 0.2% Triton X‐100 was added to permeabilize the cells. All samples were sealed with Rapid Sealing Solution (Mishushengwu, China). Primary antibodies (anti‐ALP, anti‐COLIA1, anti‐RUNX2, anti‐OPN, anti‐BSP, anti‐CD86, anti‐CD206, anti‐INOS, anti‐Arg‐1) were then separately added to each well and incubated overnight at 4 4 °C. After three rinses with PBS, the corresponding fluorescent secondary antibodies (BOSTER, China) were added for cultivation in the dark for 1 h. All samples were counterstained with DAPI and observed under a laser confocal microscope.

### Apoptosis Assay

Apoptosis assay was employed utilizing Annexin V‐FITC/PI kit(V13242, Thermo Fisher Scientific, USA)following the instruction. In brief, RAW264.7 inoculated into 48‐well plates were co‐cultured with different samples. After incubation for 5 days, cells were digested using trypsin and resuspended into binding buffer. Then add 5 µL Annexin V‐FITC and 5 µL PI. Cell apoptosis was analyzed through flow cytometry. The gating setup was based on FMO control.

### Flow Cytometry

The RAW264.7 cells were co‐cultured with different samples in an incubator at a constant temperature of 37 °C for a period of 5 days. The RAW264.7 cells of each group were digested and centrifuged, and the supernatant was discarded. Then the cells were divided into two parts. One part of the cells was resuspended with 200 µL of PBS buffer, and the cells were incubated for 30 min at 4 °C with the addition of CD86 (0.25 µg mL^−1^) to each flow‐through tube. The other CD206 was incubated for 30 min at 4 °C with the addition of CD206 (0.5 µg dose^−1^) to break the membrane before hatching; the cells were washed twice with PBS buffer and the cells were incubated for 30 min at 4 °C. The other CD206 was incubated with CD206 (0.5 µg dose^−1^) for 30 min at 4 °C after membrane rupture and then incubated for 30 min at 4 °C.

### In Vitro Antibacterial Activity

Staphylococcus aureus (S. aureus, ATCC 25923) and Escherichia coli (E. coli, ATCC 25922) were used for the antibacterial assay and were grown in Luria‐Bertani (LB) medium (Luqiao, China). The bacteria were then picked with the inoculation loop and dissolved in the LB medium to obtain the bacterial suspensions, which were then diluted with sterile PBS to a concentration of 1 × 10^7^ per mL. 500µL of each bacterial suspension was seeded onto different samples in a 24‐well plate and statically incubated at 37 °C for 12 h to observe the morphology of the cultured bacteria. Subsequently, different samples were dried under vacuum, sputtered with gold, and morphologically observed by FE‐SEM (JSM‐7900F, Japan) The antimicrobial efficacy was determined by plate count. The bacterial eluent in different groups was diluted with sterile PBS to the appropriate concentration, and then 100 µL of the diluted bacterial eluent was spread onto LB agar plates and incubated at 37 °C for 24 h. Then, the number of colony‐forming units (CFU) was counted and imaged, and the antibacterial ratio was determined by the following formula:

(2) $$\mathrm{Antibacterial}\;\mathrm{ratio}=\frac{A-B}{A}$$

where A referred to average control CFUs and B referred to average CFUs from different experimental groups.

### In Vivo Modeling

Fifty Sprague‐Dawley rats (SD, male, 5 weeks old, mean weight = 250 ± 30 g) were randomized into five groups:TC4, Ta‐Ag, Ag/Ta, Ta/Ag, and Blank control group (10 animals per group). The protocol for the animal experiments was approved by the animal ethics committee of Xi'an Jiaotong University(NO.XJTUAE2023‐2333). S. aureus (2 × 10^3^ bacteria in 20 µL PBS) was evenly coated on different implants, and then the implants were placed in a moist environment at 37 °C for 4 h to allow bacterial adhesion. All experimental rats were then given two implants in the distal femur under aseptic conditions. Briefly, SD was anaesthetized by intraperitoneal injection of 2% pentobarbital (2ml Kg^−1^). The extensor mechanism was then dislocated laterally with the patella by making a 10 mm longitudinal incision along the medial aspect of the knee joint. The knee was flexed and a bone tube was drilled from the intercondylar notch using low‐speed handpieces. Normal saline was used throughout the procedure for cooling and irrigation. Different groups of implants were then inserted through the distal femoral epiphysis into the femoral canal until one end of the implant meets the femoral canal until the implant end was below the joint surface. Then the patella was repositioned. Finally, analgesics were administered in the drinking water of the experimental rats for 3 days.

At 4 weeks and 8 weeks after surgery, the SD was sacrificed by intravenous overdose of pentobarbital, and the tissue samples were harvested and photographed. Then, the femurs with for the following procedures; First, A cylinder with a diameter of 200 µm and a length of 4 mm, located near the growth femoral growth plate, was defined as the volume of interest for micro‐CT (AX‐2000, Ningbo, China) analysis. 3D image reconstruction was performed and morphometric parameters including bone volume/total volume (BV/TV), trabecular number (Tb⋅N), and trabecular separation (Tb.Sp) were systematically evaluated.

### Histological and Bone‐Implant Contact Evaluation

Immobilization of implanted femurs in 4% phosphate formalin for 48 h and dehydration in a series of graded ethanol (70–100%, v/v) d. The specimens were sectioned perpendicular to the long axis of the implants at a thickness of 150µm using a Leica SP1600 microtome (Leica, Germany). They were polished to a thickness of 20µm. Van Gieson's picrofuchsin (VG) and Methylene blue staining were used to reveal the mineralized bone tissue, which appeared as red or blue. At the same time, histological analysis was performed using a light microscope, and the implant‐bone contact (BIC) index was determined by histometric analysis (BIOQUANT OSTEO, USA).

### Statistical Analysis

Values were presented as mean ± standard deviation and were calculated using GraphPad Prism9.4 (San Diego, CA). All experiments were repeated at least three times. Tests between two groups were performed using unpaired two‐tailed Student's t‐test, and multiple comparisons were performed using nested one‐way ANOVA with Tukey's post‐hoc analysis. Data were presented as mean ± standard deviation (SD). A difference was considered statistically significant at ^*^ *p* < 0.05.

## Conflict of Interest

The authors declare no conflict of interest.

## Author Contributions

F.X., G.Z., Y.G., and S.N. conceived the study. F.X. and Y.G. designed the experiments. F.X., G.Z., Y.G., X.L., M.Y., H.C. and L.X. performed all experiments. Y.G., X.S., N.Z., X.Z. and K.Q. analyzed the data. F.X., Y.G., B.L., J.T. and S.N. wrote the manuscript. All authors revised the manuscript.

## Supporting information



Supporting Information

## Data Availability

The data that support the findings of this study are available from the corresponding author upon reasonable request.
