# Supplementary material for: Enhancement of Osseointegration via Endogenous Electric Field by Regulating the Charge Microenvironments around Implants
Source: Adv Healthc Mater. 2025 Jan 5;14(6):2403388. doi: 10.1002/adhm.202403388 (PMC11874649; doi:10.1002/adhm.202403388)
Supplement: Supplementary file 1 — Supporting Information [file ADHM-14-0-s001.docx]

**Figure S1(A):** The SEM observation on cross section of samples. **(B):** The Hardness and Young’s modulus of samples.


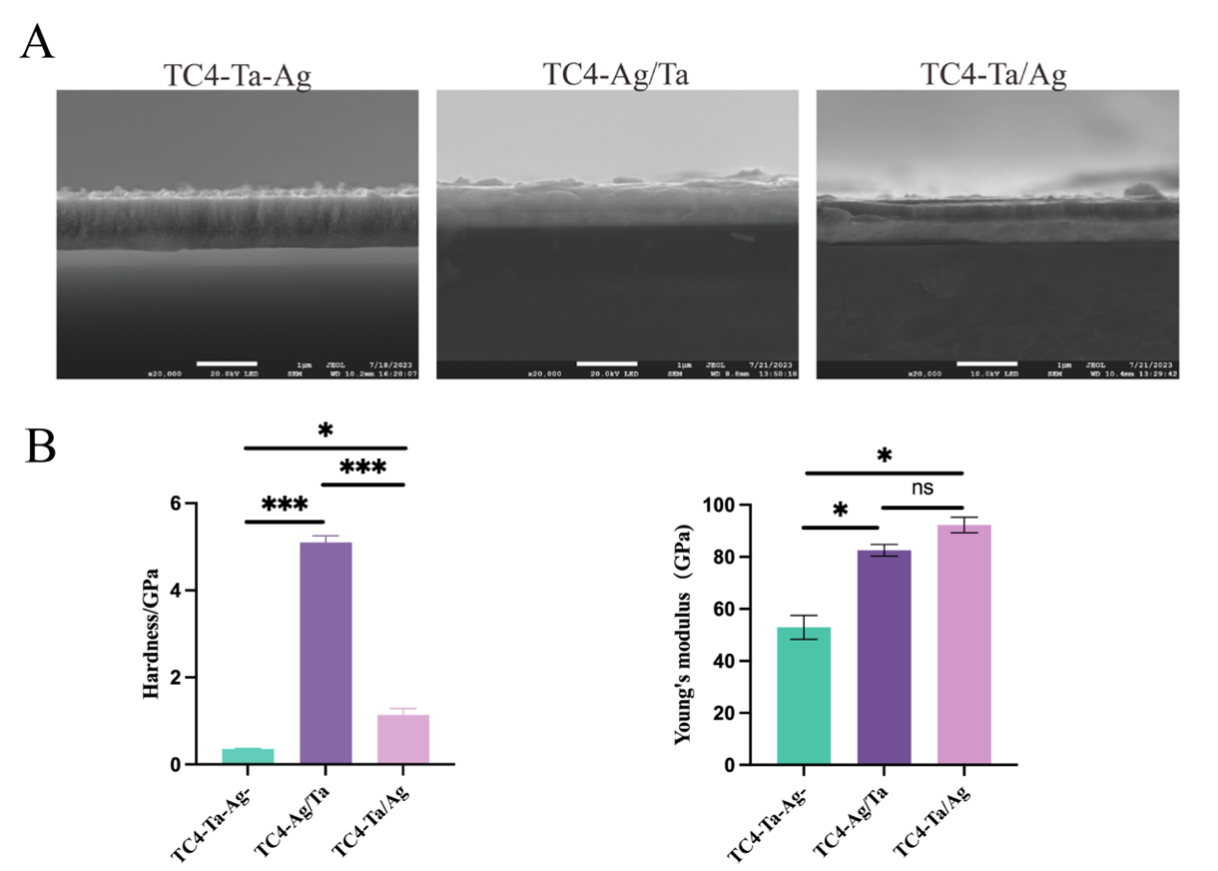


**Figure S2(A):**Living/dead staining of rBMSCs on different groups (Scale bar = 200 μm). **(B)**: Cytoskeleton staining of adhered rBMSCs on different groups (Scale bar = 100 μm).

**
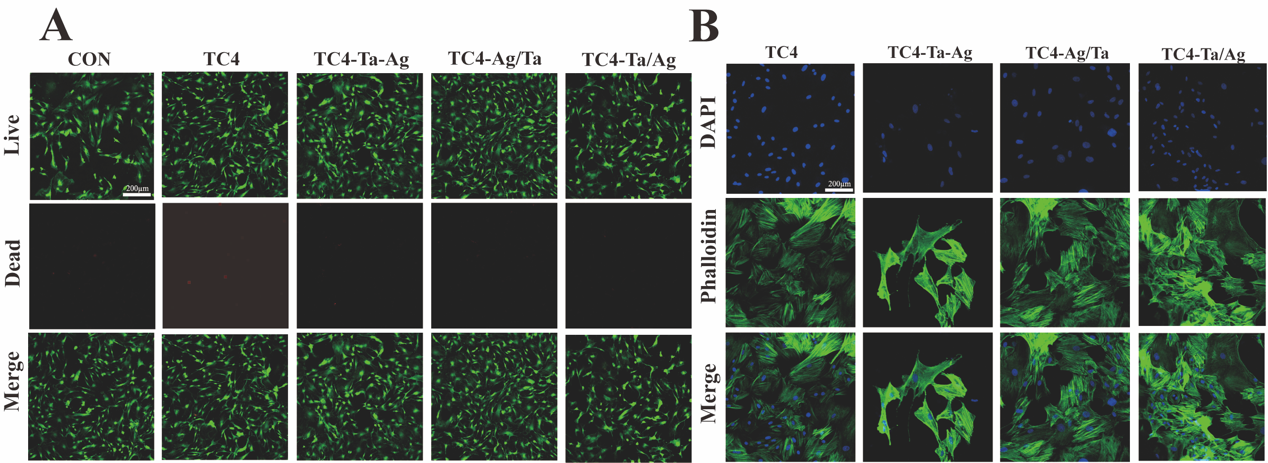
**

**Figure S3(A、B)**: The effect of apoptosis of RAW264.7 after inoculation on different groups surface was evaluated using apoptosis assay.**(C、D)**: Effect of inoculation on RAW264.7 protein expression (CD86、CD206、INOS、Arg-1)on the surface of different groups using WB. **(E、F)**: Effect of inoculation on RAW264.7 protein expression (CD86、CD206、INOS、Arg-1)on the surface of different groups using immunofluorescence.


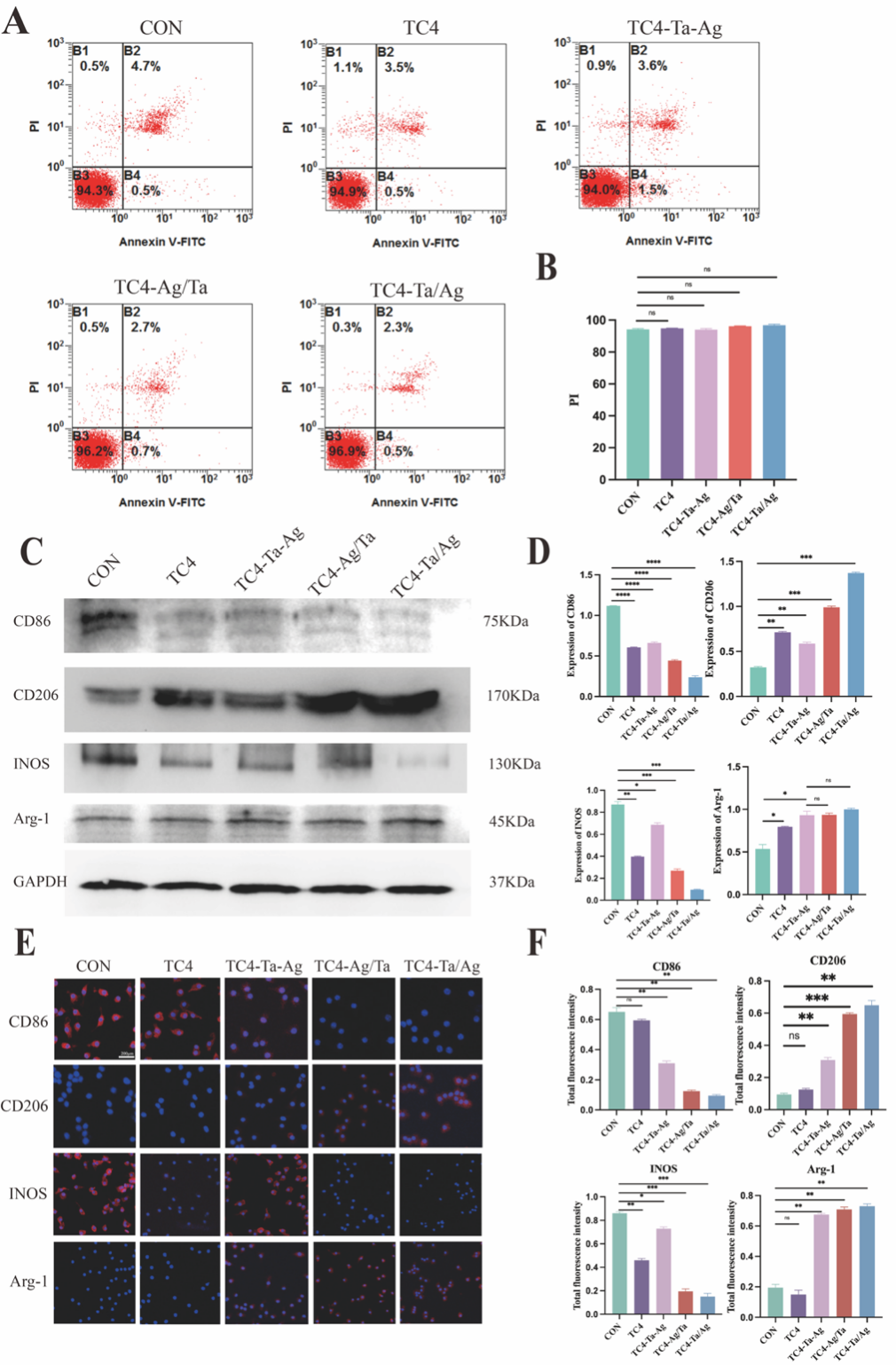


**Figure S4**: Living/dead staining of E.coli and S.aureus on different groups (Scale bar = 100 μm).


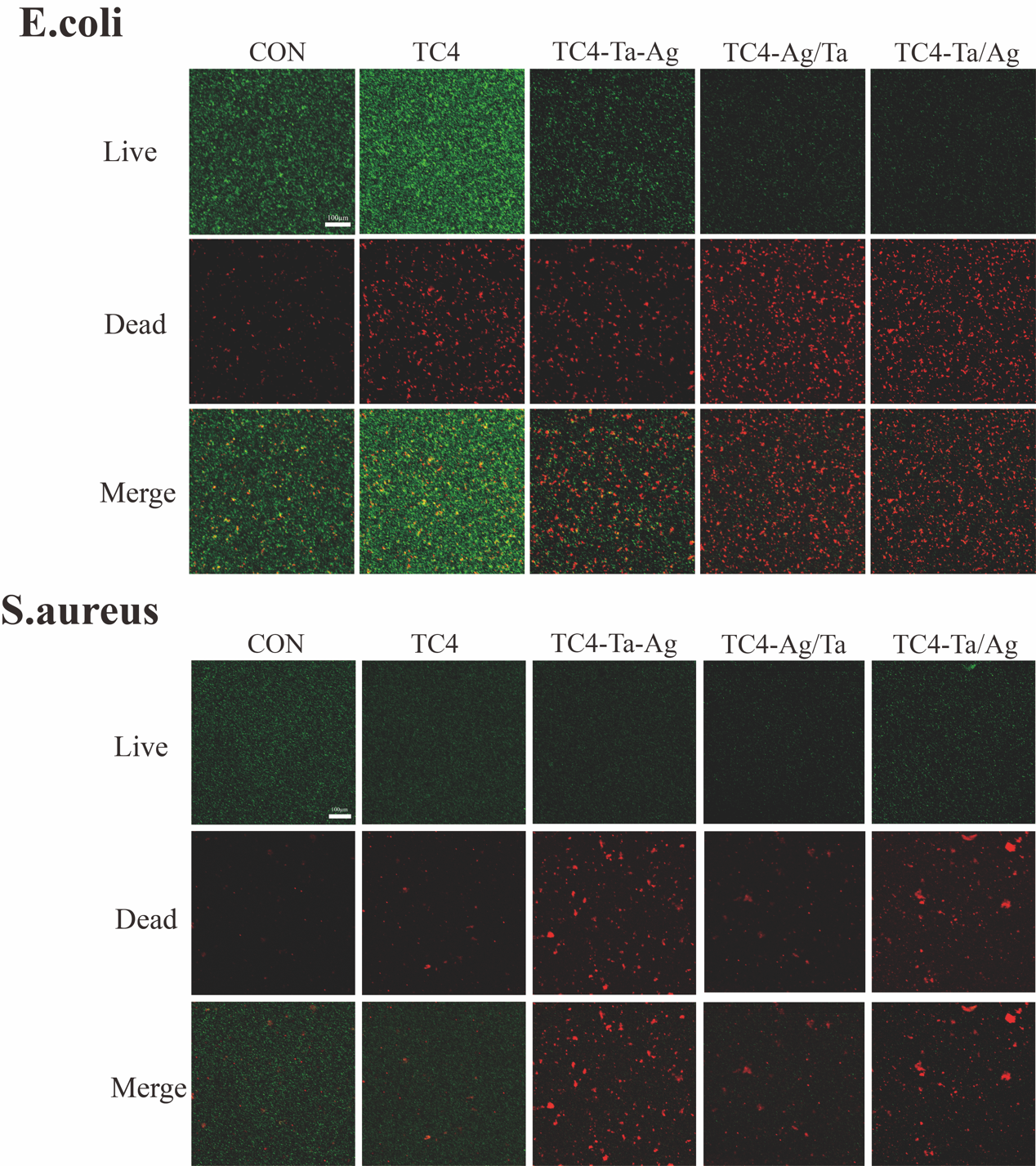


**Figure S5**: HE staining of the heart, liver, spleen, lungs, kidneys, and testes on different groups.


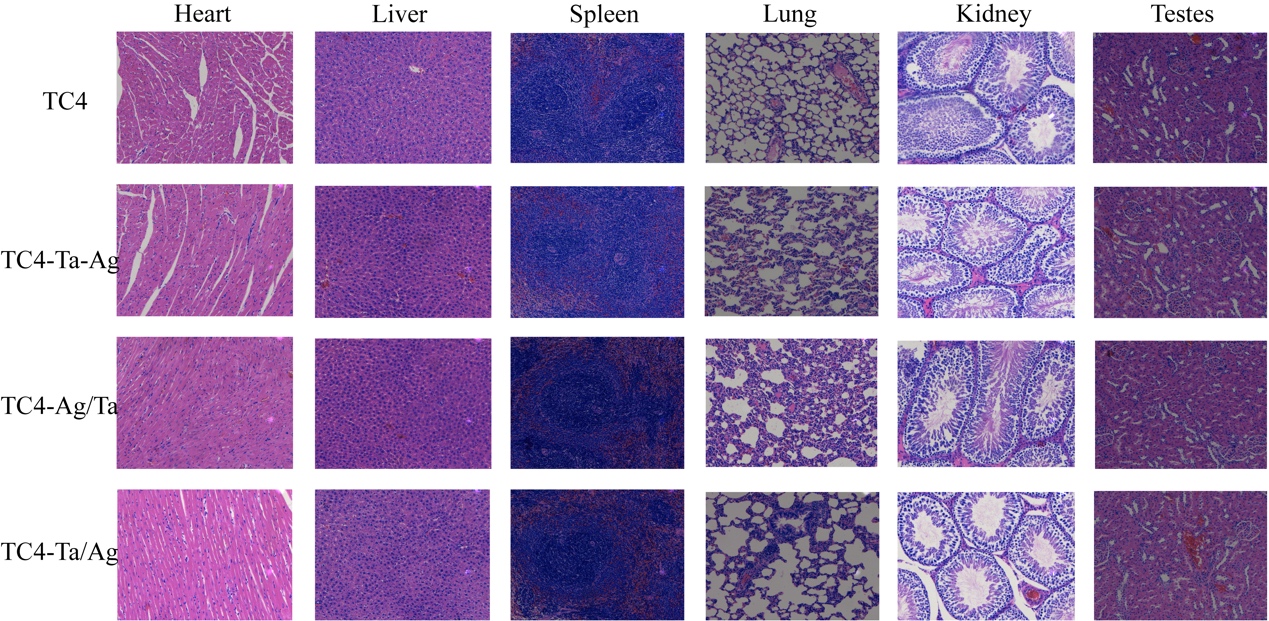


Table S1 Quantitative of elemental composition of the samples

| Samples | O(Wt%) | Ta(Wt%) | Ag(Wt%) | Total(Wt%) |
| --- | --- | --- | --- | --- |
| TC4-Ta-Ag | 2.46±0.1715 | 37.36±1.396 | 60.19±0.42 | 100 |
| TC4-Ag/Ta | 2.87±0.1325 | 64.48±1.423 | 32.66±0.397 | 100 |
| TC4-Ta/Ag | 0.9±0.1418 | 12.12±1.296 | 86.99±0.451 | 100 |

Table S2 Primer Sequence

| GENE | Primer Sequence（5’- 3’） |
| --- | --- |
| ALP | F:5′-ATGTCTGGAACCGCACTGAAC-3′  R:5′-AGCCTTTGGGATTCTTTGTCAG-3′ |
| RUNX2 | F:5′-CATGGCCGGGAATGATGAG-3′  R:5′-TGTGAAGACCGTTATGGTCAAAGTG-3′ |
| COLIA1 | F:5′-GACATGTTCAGCTTTGTGGACCTC-3′  R:5′-GGGACCCTTAGGCCATTGTGTA-3′ |
| OPN | F: 5′-GCCGAGGTGATAGCTTGGCTTA-3′  R: 5′-TTGATAGCCTCATCGGACTCCTG-3′ |
| BSP | F:5′-AGCTGACCAGTTATGGCACC-3′  R:5′-TTCCCCATACTCAACCGTGC-3′ |
